# Bisphosphonate-Induced Periprosthetic Fracture: A Cause of Painful Total Hip Arthroplasty

**DOI:** 10.1155/2014/631709

**Published:** 2014-12-10

**Authors:** Rahul Bhattacharyya, Stephanie Spence, Gavin O'Neill, Kumar Periasamy

**Affiliations:** ^1^ST2 Trauma and Orthopaedics, West of Scotland Deanery, Glasgow, UK; ^2^ST3 Trauma and Orthopaedics, West of Scotland Deanery, Glasgow, UK; ^3^Hairmyres Hospital, NHS Lanarkshire, Eaglesham Road, East Kilbride G75 8RG, UK

## Abstract

*Background*. Cases have been reported in the literature of periprosthetic fractures associated with the use of bisphosphonates occurring in the long term following a Total Hip Replacement (THR). We report an interesting case of periprosthetic fracture secondary to bisphosphonate use only a few months after a THR. *Case Report*. A 72-year-old lady (on bisphosphonates for 10 years) underwent a THR for osteoarthritis. She was pain-free in the first four months postoperatively. Thereafter, she developed spontaneous onset of pain in the lateral aspect of her thigh and groin and found it difficult to weight-bear. X-rays and blood tests were unremarkable. An ultrasound and MRI scan showed no evidence of effusion/collection or periprosthetic fracture. A radionuclide bone scan showed an abnormal appearance of the right femoral shaft. A subsequent CT scan showed an oblique vertical split on the anterior surface of the upper right femoral shaft. This stress fracture was managed nonoperatively with protected weight bearing. She has progressed well with good clinical and radiological signs of fracture healing. *Conclusion*. This case is an important addition to our knowledge that bisphosphonate-induced periprosthetic stress fractures can be a cause of hip pain only a few months following a THR.

## 1. Introduction

Bisphosphonates are osteoclast inhibitors used to treat osteoporosis and other metabolic bone diseases [1–5]. Although they have reduced the incidence of osteoporotic fractures, there is an increased risk of subtrochanteric and femoral shaft fractures amongst patients on long-term bisphosphonates [6]. There have been cases reported in the literature of periprosthetic fractures associated with the use of bisphosphonates occurring in the long term following a Total Hip Replacement (THR) [7, 8]. We report a very interesting case of a 72-year-old lady who had thigh and groin pain only four months after a routine THR and was eventually found to have a periprosthetic fracture after a series of investigations. This case is an important addition to our knowledge that bisphosphonate-induced periprosthetic fractures should be on the orthopaedic surgeon's differential diagnosis as an explanation of pain following recent arthroplasty surgery.

## 2. Case Presentation

**Figure 1:**
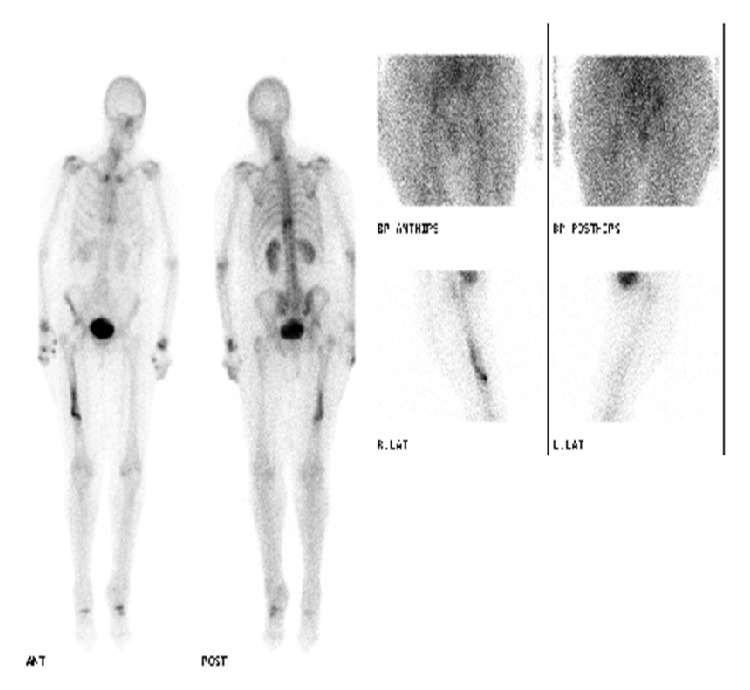
Radionuclide bone scan showing increased activity in right femoral shaft.

**Figure 2:**
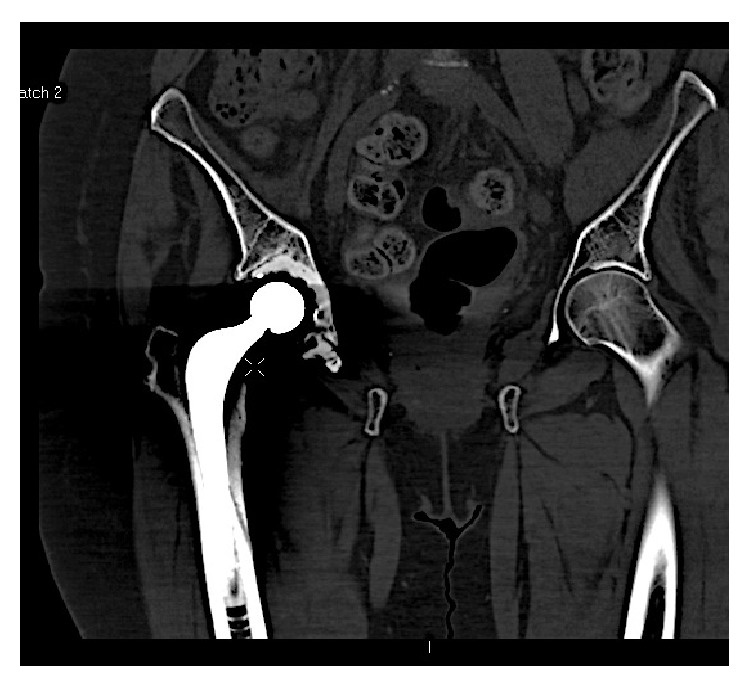
CT scan showing vertical split in right femoral shaft.

**Figure 3:**
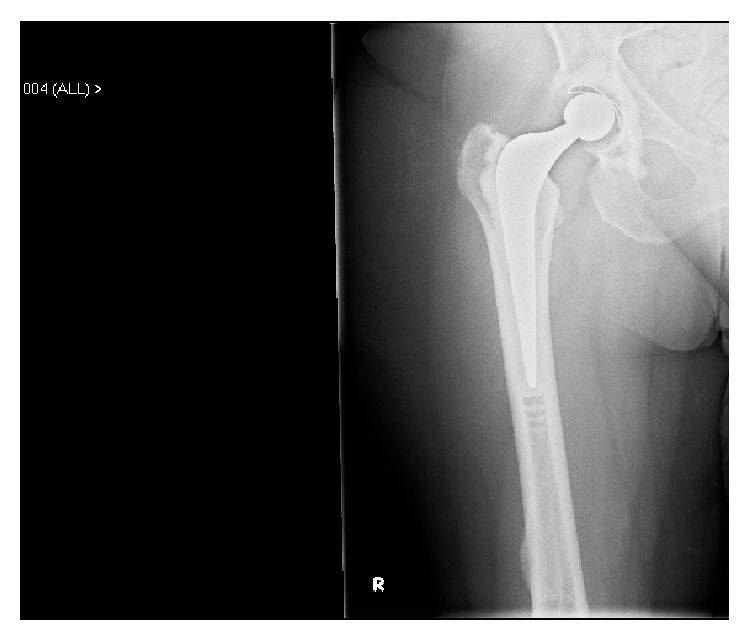
X-ray right femur showing callus formation.

A 72-year-old lady presented to us with osteoarthritis of her right hip in July 2012. She had a past medical history of rheumatoid arthritis for 20 years, Parkinson's disease, chronic anaemia, and osteoporosis. She was on alendronic acid for osteoporosis for 10 years. Other medications included Madopar, methotrexate, sulfasalazine, prednisolone, Adcal D3, aspirin, and bisoprolol. She was a nonsmoker and did not drink any alcohol. The patient underwent a routine cemented THR in October 2012. She had no complications in the perioperative period and was pain-free in the first four months following the procedure. Thereafter, she developed spontaneous onset of pain in the lateral aspect of her thigh, buttock, and groin and found it difficult to weight-bear. Plain X-rays and blood tests including inflammatory markers performed at this stage were unremarkable. The initial impression was infection, abductor dysfunction, or referred pain from the back. An outpatient ultrasound (US) scan of her right hip was organised. Whilst waiting for this scan she had an episode of pain and felt a crack in her thigh whilst turning in bed at night in June 2013 (eight months after her THR) and was subsequently unable to weight-bear. She was admitted to hospital and plain X-rays of her pelvis, right hip, and femur and blood tests were all normal. The US scan was normal and an MRI scan performed at this stage showed no evidence of effusion/collection or periprosthetic fracture. Plain X-rays of her right femur repeated again in 2 weeks did not show any abnormality. A radionuclide bone scan was performed at this stage, which showed an abnormal appearance of the right femoral shaft, which could indicate infection or a fracture (refer to Figure 1). A CT scan was then performed focusing on the area of the hot spot, which showed an oblique vertical split on the anterior surface of the upper right femoral shaft (refer to Figure 2). Therefore a diagnosis of stress fracture secondary to her long-term bisphosphonate use was made. This was managed nonoperatively with protected weight bearing and the bisphosphonates were stopped. She has progressed well with good clinical and radiological signs of fracture healing (refer to Figure 3) seen during her follow-up clinic visit in September 2013.

## 3. Discussion

This case highlights bisphosphonate-induced periprosthetic femoral stress fracture as a recognisable cause for a painful THR in the early months following a THR. Although not common, it is important to consider this as part of the differential diagnosis of pain following THR especially in a population where bisphosphonates are commonly used for the treatment of osteoporosis. A case reported [7] in literature described an 81-year-old lady who had bilateral hybrid THRs and presented with activity related pain in her left thigh approximately 8 years after her procedure. After thorough investigation she was found to have a periprosthetic fracture around a well fixed implant. She was treated nonoperatively with protected weight bearing and a course of teriparatide for six months with good recovery. Another study [8] reports three cases of bisphosphonate-induced periprosthetic fractures three years, 15 years, and 18 years, respectively, following the initial operation. All cases required a series of investigations to diagnose the fracture and they were successfully managed nonoperatively with protected weight bearing and teriparatide/calcium supplements. Sayed-Noor and Sjödén [9] reported that when a cemented implant is well fixed, the load is shared through the implant, the cement, and the bone. Proximally, the majority of the load is borne through the implant and cement, and the load shared by the bone gradually increases from proximal to distal, with the load fully borne by the bone around the tip of the cement. Theoretically, most of the load around the middle and distal thirds of the stem should be borne through the implant and cement and not the lateral cortical bone, even in a severely osteopenic patient. A fracture at the distal tip of the cement or the tip of a noncemented femoral component may not be unexpected. In all the cases reported above the periprosthetic fracture occurred much later after the initial operation. Thus far, there have been no cases reported in literature showing bisphosphonate-induced periprosthetic fracture as a cause of painful THR in the short term (within 6 months) after the surgery. Our case highlights the fact that this can be a cause for a painful THR in the short term and should be considered to avoid unnecessary delays in diagnosis and treatment.

## References

[1] Liberman U. A., Weiss S. R., Bröll J., Minne H. W., Quan H., Bell N. H., Rodriguez-Portales J., Downs R. W., Dequeker J., Favus M., Seeman E., Recker R. R., Capizzi T., Santora A. C., Lombardi A., Shah R. V., Hirsch L. J., Karpf D. B. (1995). Effect of oral alendronate on bone mineral density and the incidence of fractures in postmenopausal osteoporosis. *The New England Journal of Medicine*.

[2] Tonino R. P., Meunier P. J., Emkey R. (2000). Skeletal benefits of alendronate: 7-Year treatment of postmenopausal osteoporotic women. *The Journal of Clinical Endocrinology & Metabolism*.

[3] Black D. M., Thompson D. E., Bauer D. C. (2000). Fracture risk reduction with alen—dronate in women with osteoporosis: the fracture intervention trial. *The Journal of Clinical Endocrinology and Metabolism*.

[4] Orwoll E., Ettinger M., Weiss S., Miller P., Kendler D., Graham J., Adami S., Weber K., Lorenc R., Pietschmann P., Vandormael K., Lombardi A. (2000). Alendronate for the treatment of osteoporosis in men. *The New England Journal of Medicine*.

[5] Bush L. A., Chew F. S. (2008). Subtrochanteric femoral insufficiency fracture following bisphosphonate therapy for osseous metastases. *Radiology Case Reports*.

[6] Gedmintas L., Solomon D. H., Kim S. C. (2013). Bisphosphonates and risk of subtrochanteric, femoral shaft, and atypical femur fracture: a systematic review and meta-analysis. *Journal of Bone and Mineral Research*.

[7] Cross M. B., Nam D., Van Der Meulen M. C. H., Bostrom M. P. G. (2012). A rare case of a bisphosphonate-induced peri-prosthetic femoral fracture. *Journal of Bone and Joint Surgery—Series B*.

[8] Curtin B. M., Fehring T. K. (2011). Bisphosphonate fractures as a cause of painful total hip arthroplasty. *Orthopedics*.

[9] Sayed-Noor A. S., Sjödén, G. O. (2009). Case reports: two femoral insufficiency fractures after long-term alendronate therapy. *Clinical Orthopaedics and Related Research*.

